# Lagged Effect of Diurnal Temperature Range on Mortality in a Subtropical Megacity of China

**DOI:** 10.1371/journal.pone.0055280

**Published:** 2013-02-06

**Authors:** Yuan Luo, Yonghui Zhang, Tao Liu, Shannon Rutherford, Yanjun Xu, Xiaojun Xu, Wei Wu, Jianpeng Xiao, Weilin Zeng, Cordia Chu, Wenjun Ma

**Affiliations:** 1 Department of Environment and Health, Guangdong Provincial Institute of Public Health, Guangzhou, Guangdong, China; 2 Center for Disease Control and Prevention of Guangdong Province, Guangzhou, Guangdong, China; 3 Center for Environment and Population Health, School of Environment, Griffith University, Brisbane, Queensland, Australia; University of Ottawa, Canada

## Abstract

**Background:**

Many studies have found extreme temperature can increase the risk of mortality. However, it is not clear whether extreme diurnal temperature range (DTR) is associated with daily disease-specific mortality, and how season might modify any association.

**Objectives:**

To better understand the acute effect of DTR on mortality and identify whether season is a modifier of the DTR effect.

**Methods:**

The distributed lag nonlinear model (DLNM) was applied to assess the non-linear and delayed effects of DTR on deaths (non-accidental mortality (NAD), cardiovascular disease (CVD), respiratory disease (RD) and cerebrovascular disease (CBD)) in the full year, the cold season and the warm season.

**Results:**

A non-linear relationship was consistently found between extreme DTR and mortality. Immediate effects of extreme low DTR on all types of mortality were stronger than those of extreme high DTR in the full year. The cumulative effects of extreme DTRs increased with the increment of lag days for all types of mortality in cold season, and they were greater for extreme high DTRs than those of extreme low DTRs. In hot season, the cumulative effects for extreme low DTRs increased with the increment of lag days, but for extreme high DTR they reached maxima at a lag of 13 days for all types of mortality except for CBD(at lag6 days), and then decreased.

**Conclusions:**

Our findings suggest that extreme DTR is an independent risk factor of daily mortality, and season is a modifier of the association of DTR with daily mortality.

## Introduction

Climate change, represented by a general increase in both mean temperature and temperature variability, has been observed over the last half century [1]. The impact of climate change or variability on humans has long been a matter of public health interest [2], [3]. Typically, J-, V-, or U-shaped associations between temperature and mortality risk have been observed in many studies in the last two decades [4], [5]. Recent studies have explored the effects of different temperature indicators such as mean, minimal and maximal temperature, mean, minimal and maximal apparent temperature, humidex, and diurnal temperature range (DTR) on health [6]–[8].

DTR is a meteorological indicator associated with the global climate change and urbanization. It is defined as the difference between maximal and minimal temperatures within 1 day. In most urban regions of the world, DTR is decreasing because nocturnal minimal temperatures have risen faster than daytime maximal temperatures in the context of global climate change [9], [10]. For example, the DTR in Guangzhou city of southern China decreased 1.71°C from 1960 to 2005, which was much larger than the global average DTR decrease rate of around 0.07°C per decade from 1950 to 2004 [11], [12]. Hence in order to reflect climate change, DTR may be a better indicator when analyzing temperature impacts on human health.

However there are only a few published studies investigated DTR-related health effects and they focused on the effects of high DTR, reporting a linear relationship between higher DTR and mortality [13]–[15]. Whether a low DTR is associated with mortality and how season modifies the effects of DTR on mortality are still unclear and hence more studies are needed to more comprehensively explore the effects of DTR on health.

Most previous studies of the associations of various temperature indicators and mortality focused on the same day effects of temperature [16], [17] with a few considering about the lag effects [18]. Moreover, these have used single-day models to measure the moving average value’s lag effects [19], which may overestimate the effects of current-day exposure by overlapping with risks from exposures in previous days [9], and cannot adequately capture the total effect of exposure on mortality [20], [21]. Recently, a distributed lag non-linear model (DLNM) was developed to describe simultaneously the potentially non-linear relationship in the space of temperature and along the lag days [22]. The DLNM is a more flexible and biologically plausible method to quantify individual lags, especially at short lag times. Furthermore, mortality displacement may also be observed from the lag effect structure estimated by DLNM, which may enhance our understanding of the effects of temperature on mortality [21], [23], [24]. Hence DLNM was considered a more appropriate method to capture the total effect of temperature on mortality for this study.

In the current study, we used DLNM to conduct a time-series study in Guangzhou, China to examine the acute effect of DTR on mortality, and further investigate the modification of season on the effect. We hypothesized that extreme DTRs would result in more serious adverse impacts on mortality than moderate DTRs.

## Materials and Methods

### Study Site

**Figure 1:**
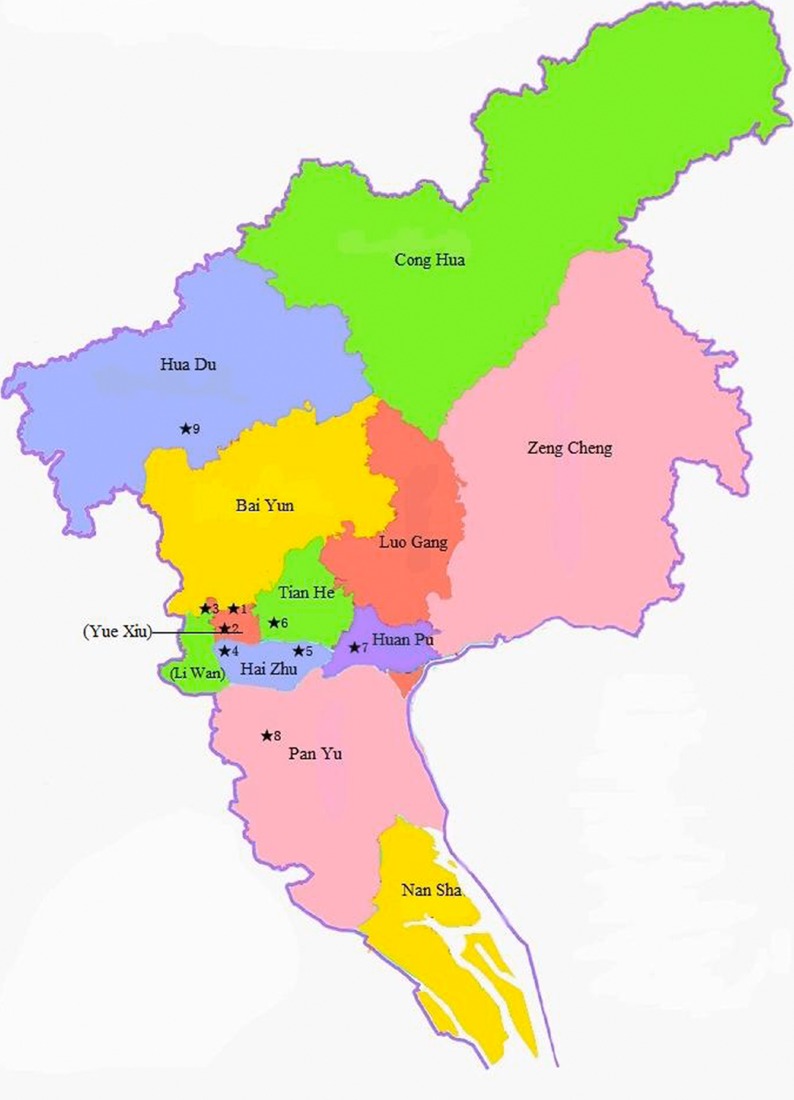
District map of Guangzhou, China, with monitoring station locations. Two districts (the district names) were included in the present study.

Guangzhou is located in South China and in 2010 it had a population of 11.1 million. It has a typical sub-tropical, humid, monsoon climate, with average annual temperature of 22°C and average rainfall of 1500–2000 mm. Due to data availability, mortality data was obtained for two districts (Yue Xiu and Li Wan) of Guangzhou. In 2010, there were a total of 1.9 million residents in these two districts, representing 17.1% of Guangzhou’s population. Fig. 1 presents a district map of Guangzhou showing the locations of the nine automatic, online air pollution monitoring stations.

### Data

Daily meteorological data for YueXiu and LiWan districts were obtained from Guangdong Meteorological Bureau for the January 1^st^ 2006 to December 31^st^ 2008 periods. Parameters collected included minimum temperature (tmin), maximum temperature (tmax), mean temperature (tm), relative humidity (rh), mean wind speed (ws), mean precipitation (pre), mean dew point temperature (dew) and mean pressure (p). DTR was calculated as the difference between maximal and minimal temperatures within 1 day. In order to analyze the effects of extreme DTRs, we defined DTRs lower than the 1^st^ percentile (1.7°C) and higher than the 99^th^ percentile (14.5°C) of DTRs as extreme low and extreme high DTRs in the current study.

Daily non-accidental mortality data covering the same period was obtained from the Center for Disease Control and Prevention of Guangdong Province (GDCDC) for the same districts. The original source of information on mortality was the death certificate which included the age and sex of the deceased and the date and causes of death. The causes of death excluded accidental deaths and were coded according to International Statistical Classification of Diseases and Related Health Problems 10th Revision (ICD-10) [25]. In addition to NAD, the mortality data were classified into deaths due to RD (ICD-10: J00–J99), CVD (ICD-10: I00–I99) and CBD (ICD-10: I60–I69).

Daily 24 hours average air pollutant data were collected from Guangzhou Environmental Monitoring Center, averaging the value from number 1–3 monitoring stations in two selected districts for the same time period (Figure 1). Air pollution data included particular matter less than or equal to 10 µm (PM_10_), sulfur dioxide (SO_2_) and nitrogen dioxide (NO_2_).

### Statistics Analysis

As counts of daily mortality data typically follow a Poisson distribution, DLNM with a log link and Poisson error was used to analyze the effect of DTR on daily mortality and its associated lag structure. This method can account for smooth fluctuations in daily mortality to overcome the serial correlation of time-series data [22]. Moreover, in order to observe the independent effects of DTR on mortality outcomes, we controlled for potential confounders such as long-term trend of daily mortality, day of week effect, mean temperature, humidity and air pollutants. We used the following model:
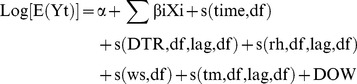
(1)Where the subscript t denotes the time (day) of the observation; E(Yt) denotes the expected number of deaths at day t; α is the intercept; X is the independent variable for the linear effect on the dependent variable, here indicating the pollutant concentrations of SO_2_, NO_2_ and PM_10_; β is the coefficient for the independent variable in the regression model; s() is cubic regression spline function for the non-linear variables, including time, DTR, mean temperature, relative humidity and wind speed; The smooth term of time is used to control for secular trends and seasonality confounding; DOW is day of week, which is a categorical variable.

To choose the degree of freedom for the non-linear variables, we used Akaike’s Information Criterion (AIC) for quasi-Poisson models [22], [26]. Finally, we chose the degree of freedom (df) as 8/year for time to remove the secular trends. The degrees of freedom (df) for mean temperature, relative humidity and wind speed were 3, and the degree of freedom for both DTR and lag in cubic regression spline function were 5. We used lags up to 27 days according to the previous study to capture the total DTR effect [27]. Previous studies found that mean temperature and relative humidity both significantly affect mortality [17]. Therefore, sensitivity analyses were conducted by changing lag structures for mean temperature and relative humidity up to the previous 2 weeks to observe the effects of DTR. The lag structures up to 4 days (lag4) were finally selected to control for mean temperature and relative humidity respectively because the DTR effects appeared to be stable after 4 lag days (See Figure S1–S2).

With the purpose of evaluating several characteristics of the DTR-mortality relationship, we classified the causes of disease into several categories for establishing models and considered the lag effects of DTR on daily mortality when building the models. As the effect of DTR may vary between hot season (from May to October) and cold season (from November to April of the next year), we conducted analyses separately for these two seasons. Moreover, we analyzed extreme low (less than 1^st^ percentile of DTR) and high DTRs (higher than 99^th^ percentile of DTR) effects on mortality among different lag structures, respectively. Furthermore, we divided the extreme low DTR into low tmax group (tmax was less than median value of tmax (16.2°C)) and high tmin group (tmin was higher than median value of tmin (15.5°C)). The extreme high DTR was divided into high tmax group (tmax was higher than the median value of tmax (23.7°C)) and low tmin group (tmin was less than median value of tmin (7.6°C)). All the relative risk (RR) and their confidence intervals (CIs) of mortality were estimated for the DTR of 8°C as the reference. The cumulative excessive rates (CERs) were calculated by the following formula:
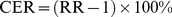
(2)


Sensitivity analysis were also performed by changing the definitions of extreme low DTRs from less than 1^st^ percentile to 2.5^th^ percentile and 5^th^ percentile, and extreme high DTRs from higher than 99^th^ percentile to 97.5^th^ percentile and 95^th^ percentile, respectively. Another sensitivity analysis was performed by including SO2 and NO2 as non-linear variables in the model.

All statistical tests were two-sided and values of P<0.05 were considered statistically significant. The DLNM packages in R software Version 2.14.0 (R Development Core Team, 2011) were used to fit all models and estimate the exact standard errors of regression coefficients.

## Results

### Data Description

Table 1 shows the characteristics of daily weather variables, mortality and air pollutants in Guangzhou for the study period. A total of 36168 non-accidental deaths were recorded during the January 1^st^, 2006 to December 31^st^, 2008 period. On average, daily NAD, CVD, RD and CBD-related deaths were 32.7, 11.4, 5.6 and 4.2, respectively. The average daily mean temperature, diurnal temperature range, relative humidity and wind speed were 23.0°C, 7.5°C, 71.1% and 1.4. Mean concentration of NO_2_, SO_2_, PM_10_ were 59.3 µg/m^3^, 47.6 µg/m^3^ and 72.0 µg/m^3^, respectively.

**Table 1:** Summary statistics of daily weather, mortality and air pollution in Guangzhou, China (2006–2008).

Variables	Minimum	1%	25%	Median	75%	99%	Maximum	Mean	SD
**Daily deaths by causes of disease**									
NAD	11.0	18.0	27.0	32.0	37.0	56.0	81.0	32.7	8.0
CVD	2.0	4.0	8.0	11.0	14.0	25.0	36.0	11.4	4.4
RD	1.0	1.0	4.0	5.0	7.0	13.0	18.0	5.6	2.6
CVB	1.0	1.0	3.0	4.0	5.0	11.0	13.0	4.2	2.2
**Weather**									
Mean Temperature(°C)	5.4	7.7	18.6	24.5	27.8	32.0	33.5	23.0	6.1
DTR(°C)	1.0	1.7	5.5	7.6	9.2	14.7	16.9	7.5	2.8
Relative humidity (%)	25.0	34.0	64.0	72.0	81.0	92.0	94.0	71.1	13.0
Wind speed	0.5	0.6	1.1	1.3	1.7	2.9	3.1	1.4	0.5
**Air pollutants (µg/m^3^)**									
NO2	13.0	17.6	35.4	49.9	76.2	157.7	199.4	59.3	32.1
SO2	2.4	4.3	22.5	40.7	65.3	150.4	289.3	47.6	33.3
PM_10_	8.3	14.0	41.3	62.9	93.7	205.4	268.6	72.0	41.7

SD = standard deviation.

### Full-year Regression Results

**Figure 2:**
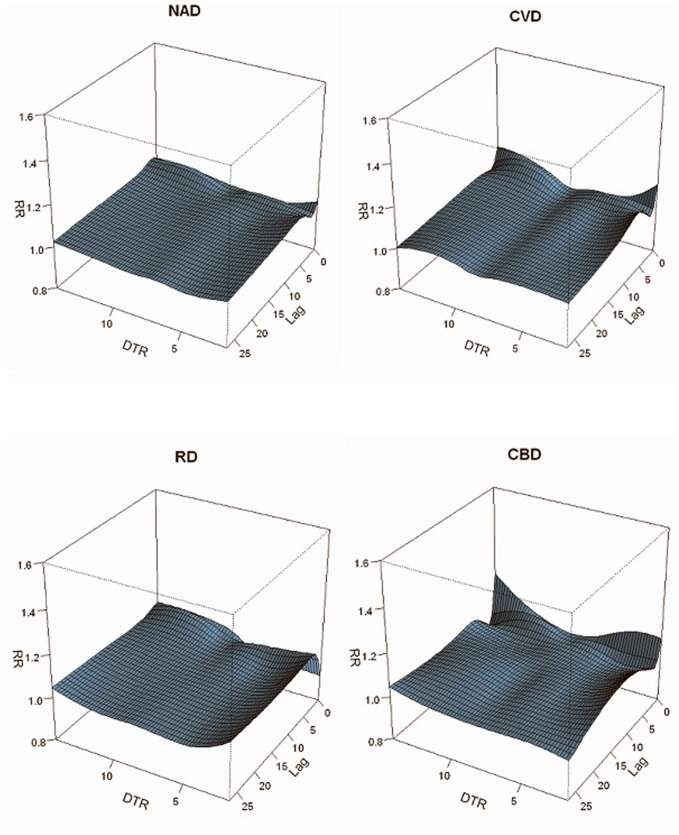
Three-Dimension plot for relative risks of mortality types by DTR.

The three-dimensional plots show the relationships between DTRs and mortality categories along the 27 lag days (Figure 2). Overall, the estimated effects of DTRs on all mortality types were non-linear, and there were significantly higher RRs for extreme DTRs compared to the DTRs of 8.0°C (the mean DTR is 7.5°C) used as a reference. Regarding extreme low DTRs, their effects on NAD, CBD and CVD-related mortality were the largest at lag0, subsequently declining for the following 2 days, and then rising again. However, it was different for RD-related mortality, where the effects were the smallest at lag0 followed by an increment for 2 days, and then declining till lag 27 days. For the extreme high DTR, its effects were the strongest at lag0 with a decline for around 10 days, and then an increase till lag 23, lag 15 and lag 27 for NAD, CVD and RD-related mortality, respectively. In terms of the CBD-related mortality, significance fluctuated along the lag days, with a maximum effect at lag0 and minimum effect at lag 5.

Table 2 shows the CERs of different DTRs on all mortality types along 27 lag days after adjustment for confounding effects from day of week, long-term trends and air pollutants. When the DTRs were extreme low or high, their CERs for all types of mortality appeared stronger than the moderate DTRs. Moreover, extreme low DTR had greater effect on all types of mortality than extreme high DTR.

**Table 2:** The CERs of different DTRs on mortality at lag0–27 in the full year.

DTR structures*	CERs (95%CI)			
	NAD (%)	CVD (%)	RD (%)	CBD (%)
1.7°C	**139.2(54.6–269.6)**	**250.7(57.0–683.3)**	**381.0(69.6–1263.8)**	**225.0(6.2–895.1)**
5.5°C	−8.3(−29.6–19.5)	49.5(−3.5–131.5)	−19.6(−54.5–41.9)	−1.6(−45.6–77.9)
7.6°C	3.3(−6.3–13.8)	15.2(−3.2–37.2)	−1.8(−20.6–21.4)	4.6(−17.3–32.3)
9.2°C	1.3(−12.1–16.7)	18.1(−2.8–43.5)	18.0(−8.9–52.8)	21.4(−7.6–59.3)
14.5°C	**124.7(44.0–250.6)**	86.0(−20.9–337.3)	207.6(−2.9–874.8)	146.0(−21.5–671.4)

* 1.7°C, 5.5°C, 7.6°C, 9.2°C and 14.5°C represent the 1^st^ percentile, 25^th^ percentile, 50^th^ percentile, 75^th^ percentile and 99^th^ percentile of DTR in Guangzhou, respectively. The 8°C of DTR was selected as the reference.

As the table 3 shows, for extreme low DTR, the cumulative effects of low maximum temperature are a little bit higher than that of high minimum temperature on all types of mortality. For extreme high DTR, the cumulative effects for both high maximum temperature group and low minimum temperature group are similar. Therefore, we did not divided extreme low or high DTR into two categories in the following results.

**Table 3:** The cumulative relative risks of extreme DTR categorized by minimum and maximum temperature on disease-specific death effects. along the 27 lag days.

Categorized DTR*	RRs (95%CI)			
	NAD	CVD	RD	CBD
Extreme low DTR – low maximum temperature	**2.587(1.636–4.091)**	**4.026(1.718–9.435)**	**6.170(2.029–18.761)**	3.222(0.953–10.890)
Extreme low DTR – high minimum temperature	**2.471(1.596–3.827)**	**3.764(1.697–8.349)**	**5.492(1.939–15.559)**	2.987(0.857–9.320)
Extreme high DTR – high maximum temperature	**2.198(1.416–3.413)**	1.873(0.804–4.365)	2.930(0.930–9.235)	2.419(0.775–7.544)
Extreme high DTR – low minimum temperature	**2.235(1.414–3.534)**	1.807(0.738–4.427)	2.944(0.873–9.923)	2.410(0.731–7.950)

Note:

* Extreme low DTR – low maximum temperature group was defined as daily maximum temperature was less than median value in the chosen extreme low DTR group (16.2°C); Extreme low DTR –high minimum temperature group was defined as daily maximum temperature was higher than median value in the chosen extreme low DTR group (15.5°C); Extreme high DTR – high maximum temperature group was defined as daily maximum temperature was higher than median value in the chosen extreme high DTR group (23.7°C); Extreme high DTR – low minimum temperature group was defined as daily minimum temperature was less than median value in the chosen extreme low DTR group (7.6°C).

### Seasonal Regression Results

**Figure 3:**
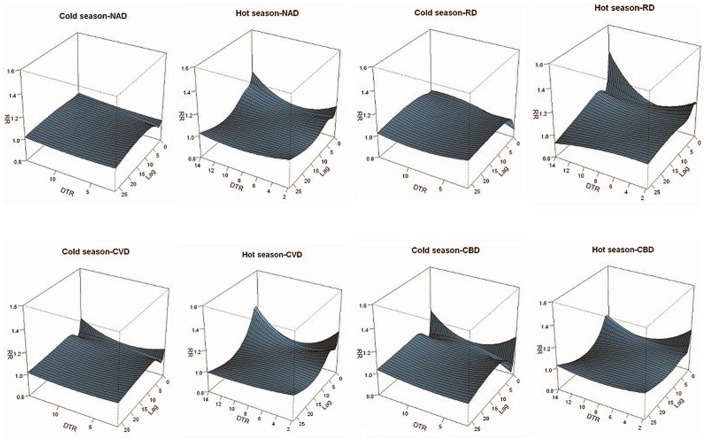
Three-Dimension plots for the effects (RR) of DTR on mortality in hot and cold season.

We further stratified the full year into hot and cold seasons to compare the effects of DTRs on mortality between different seasons. Figure 3 shows the immediate and lagged effects of DTR in hot or cold seasons along 27 day lags. The immediate effects (lag0) of extreme DTR on all types of mortality in hot season are larger than that in cold season, such as CVD-related mortality (Extreme low DTR: RR = 1.104, 95%CI: 0.943–1.292 vs. RR = 1.041, 95%CI: 0.909–1.192; Extreme high DTR: RR = 1.136, 95%CI: 0.991–1.302 vs. RR = 1.045, 95%CI: 0.899–1.215). Both the mortality effects of the extreme low and high DTRs lasted longer in cold season than in hot season, such as NAD-related mortality, in hot season, the lagged effects for extreme low and high DTR lasted till lag12 (RR = 1.001, 95%CI: 0.979–1.022) and lag 10 (RR = 1.003, 95%CI: 0.985–1.021), respectively. But in cold season, both of the lagged effects could last to lag27 (extreme low DTR: RR = 1.011, 95%CI: 0.983–1.040 vs. extreme high DTR: RR = 1.024, 95%CI: 0.993–1.056).

**Figure 4:**
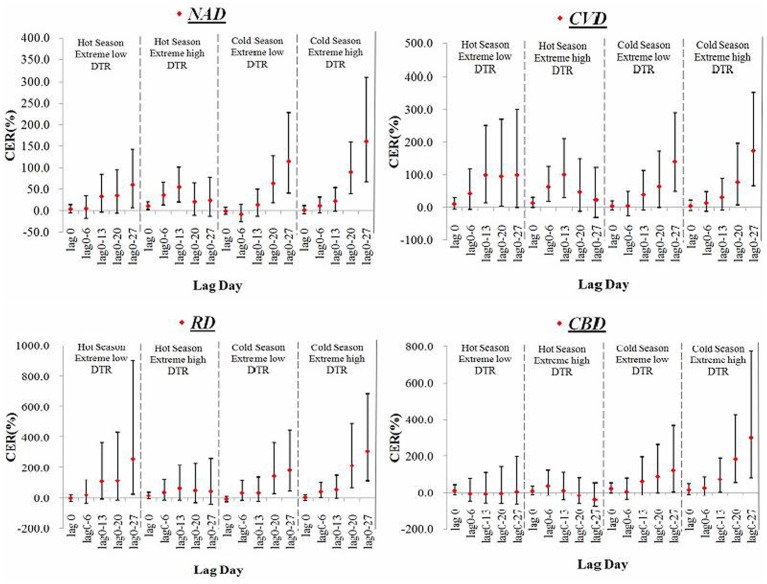
CERs of extreme low and high DTR on disease-specific mortality in hot and cold seasons at different lag days. Note: The 8°C of DTR was selected as the reference, which was regarded as minimal mortality.

In order to further estimate the overall effects of extreme DTRs on mortality in different seasons along different lag days, we compared CERs for extreme high and low DTRs in different seasons along different lag structures. Figure 4 shows the effects of extreme low and high DTR on mortality. In the cold season, the overall CERs of both extreme low and high DTR increased with the increment of lag days. However, in hot season, only the CERs for extreme low DTR increased with the lag days, and for extreme high DTR, NAD-related, CVD-related and RD-related mortality effects reached maxima after two weeks exposure (NAD: CER% = 55.4%, 95%CI: 19.9%–101.4%; CVD: CER% = 110.5%, 95%CI: 29.8%–209.7%; RD: CER% = 63.9%, 95%CI: −14.8–215.4). CBD-related mortality reached maxima after one week exposure (CBD: CER% = 36.6%, 95%CI: −15.9%–121.8%) and then decreased. Comparison of overall effects between extreme low and high DTR in different season, we found the effects of extreme low DTR were greater than extreme high DTR in hot season, but the relationship in cold season was just on the contrary.

### Sensitivity Analysis

We changed the definition of extreme high and low DTR by choosing different cut off points, which gave similar results (See Figure S3–S6) both for full year and seasonal regression analysis. Moreover, the results of the sensitivity analysis by including SO_2_ and NO_2_ as non-linear variables were also similar with the linear one (See Figure S7 and Table S1). Consequently, we believe that the methods used in this study adequately captured the main effects of DTR on mortality.

## Discussion

The acute effects of extreme temperatures (e.g., heat waves and cold spells) on daily mortality have been widely-documented in previous studies [3], [5], [22], [28]–[30]. These studies commonly used mean and maximum temperature as temperature indicators. Recently, researchers have suggested that DTR might be another important indicator to reflect the impact of temperature change on mortality and morbidity, reporting linear relationships between DTR and mortality using single-day models [31]. For example, a few previous studies examined the impact of DTR on NAD, CVD, RD and stroke mortality using a single-day model in Shanghai, and found that high DTR was significantly associated with daily mortality after adjusting for potential confounders [15], [31]–[33]. Tam et al. also observed a 1.7% increase in CVD mortality for an increase of 1°C in DTR at lag0-3 days among the elderly in Hong Kong [34].

For the present study, we used a constrained distributed lag model, employing natural splines to constrain for lag changes in coefficients, which is more appropriate to estimate the effects of short-term DTR exposure than a single-day model [9]. The results revealed that extreme DTR was significantly associated with increased mortality, which was partly consistent with previous studies that used single-day models [31], [32]. A unique finding of the current study is that the relationships between DTRs and daily mortality are nonlinear, which is similar to the previously reported temperature-mortality relationship [35]. That is, the effects of extreme high or low DTR on daily mortality were stronger than that of moderate DTR (approximately equal to mean DTR). The other novel finding of our study was that extreme low DTR was likely to have a greater effect on mortality than extreme high DTR suggesting that a small difference between minimum and maximum daily temperature is associated with a greater risk of mortality compared with a large difference in daily minimum and maximum temperature. This lack of variability within a day would possibly cause a failure of thermoregulation and result in some temperature-related morbidity and mortality. Therefore, the findings of the present study highlight the importance of not only absolute temperatures in relation to human health, but also changes in diurnal temperature, particularly extreme DTR. This has implications for understanding the impacts of climate change where in some parts of the world (e.g., central and southeastern Europe [36], North America [37] and in central and south Asia [38]) higher minimum temperatures are being predicted, suggesting a lower DTR. In a addition, urban heat island effect could also decrease DTR because the increment of the daily minimum temperature was larger than daily maximum temperature with the urbanization [10], [39].

In order to explore the health effects of extreme (e.g., very high or very low) DTRs in different seasons, the present study further stratified the entire year into hot and cold seasons. Significant associations between extreme DTR and mortality were observed in both two seasons, which further indicated that extreme DTR was a risk factor for daily mortality regardless of season. In the cold season, both of the overall effects of extreme low and high DTR increased with the increment of lag days, and the effects of extremely high DTR on all categories of mortality were greater than extremely low DTR at lag0–27 days. These results could suggest that local residents may not acclimate well to the cold season with larger change because the winter weather in Guangzhou is usually mild. Proposed mechanisms of health impact due to greater change in cold season may be associated with risk factor of human health, such as increases in blood cholesterol levels, blood pressure, plasma fibrinogen concentrations, peripheral vasoconstrictions, heart rate, platelet viscosity, and reducing the immune system’s resistance [40], [41]. These impacts of temperature change in the cold season in this normal sub-tropical area were really interesting and had implications for places that may not have thought about being affected by changes in temperature (e.g., strategies for heat extremes have been favored but possibly may need to look more closely at the cold season effects) to pay more attention to reduce the impact of DTR in cold season.

In hot season, we found that the effects of extreme low DTR on mortality increased as the lag days increased, but for extreme high DTR, the effects began to decline slowly at around lag 14 days. These findings suggest that there was some mortality displacement for extremely high DTR in the hot season within 27 lag days, which was similar to the effects of high ambient temperature found in previous studies [42], [43]. Although the real reasons for the mortality displacement in the hot seasons were not very clear, the extremely high DTR in the hot season possibly impacted the mostly frail individuals with little remaining life expectancy. However, mortality displacement was not observed for extreme low DTR in the hot season within 27 lag days. The possible reason would be that extreme low DTR, which means sustained high temperature within a day in hot season, may have a broader impact on the public because it represents a greater loss of life-years [44].

A limitation of the current study was that the data were only from one city. Therefore, it is difficult to extrapolate our results to other regions or cities. Moreover, like other studies, we used available environmental monitoring data to represent the population exposure to DTR and other covariates, which may not accurately reflect the real personal exposure to ambient temperature or air pollution due to widespread use of air-conditioners in Guangzhou, especially in the hot season.

In summary, we found that DTR was independently associated with daily mortality in Guangzhou, China. Season is a modifier of the association of DTR with daily mortality with findings being most significant for effects of extreme low DTR in hot season and for extreme high DTR in cold season. These findings highlight the importance of measuring health impacts of DTR as opposed to only conventional measures of daily temperature because DTR is projected to decrease slowly in the context of climate change and urbanization in many parts of the world.

## Supporting Information

Figure S1 **Association between DTR and mortality with adjustment of the maximum distributed lag days of mean temperature, 2006–2008.**
(TIF)

Figure S2 **Association between DTR and mortality with adjustment of the maximum distributed lag days of relative humidity, 2006–2008.**
(TIF)

Figure S3 **3-D plots for the effects of DTR on mortality in cold and hot season.** Select selected lower than 2.3°C (2.5th percentile) and higher than 13.8°C (97.5th percentile) as the extreme low and high DTRs.
(RAR)

Figure S4 **Effects of extreme low and high DTR on disease-specific deaths in hot and cold seasons at different lag times.** The 8°C of DTR was selected as the reference, which was regarded as minimal mortality; Select selected lower than 2.3°C (2.5th percentile) and higher than 13.8°C (97.5th percentile) as the extreme low and high DTRs.
(TIF)

Figure S5 **3-D plots for the effects of DTR on mortality in cold and hot season.** Notes: Select selected lower than 3.1°C (5th percentile) and higher than 12.6°C (95th percentile) as the extreme low and high DTRs.
(RAR)

Figure S6 **Effects of extreme low and high DTR on disease-specific deaths in hot and cold seasons at different lag times.** The 8°C of DTR was selected as the reference, which was regarded as minimal mortality; Select selected lower than 3.1°C (5th percentile) and higher than 12.6°C (95th percentile) as the extreme low and high DTRs.
(TIF)

Figure S7 **3-D plots for the effects of DTR on mortality in cold and hot season.** The pollution variables of SO2 and NO2 were regarded as non-linear in the DLNM. Moreover, lower than 1.7°C (1th percentile) and higher than 14.5°C (99th percentile) were selected as the extreme low and high DTRs, respectively.
(RAR)

Table S1 **The CERs of different DTRs on mortality along 27 lag days for the full year.** The type of SO2 and NO2 was considered as nonlinear in the DLNM.
(DOC)

## References

[1] WHO (2008) Protecting Health from Climate Change – World Health Day 2008. World Health Orgnization.

[2] McMicheal A, Campbell-Lendrum D, Corvalan C (2003) Climate changes and human health-risks and responses. World Health Organization, Geneva.

[3] Curriero FC, Heiner KS, Samet JM, Zeger SL, Strug L, et al (2002) Temperature and mortality in 11 cities of the eastern United States. Am J Epidemiol 155(1): 80–87. 11772788 10.1093/aje/155.1.80

[4] Braga AL, Zanobetti A, Schwartz J (2002) The effect of weather on respiratory and cardiovascular deaths in 12 U.S. cities. Environ Health Perspect 110(9): 859–863. 10.1289/ehp.02110859 PMC1240983 12204818

[5] Baccini M, Biggeri A, Accetta G, Kosatsky T, Katsouyanni K, et al (2008) Heat effects on mortality in 15 European cities. Epidemiology 19(5): 711–719. 18520615 10.1097/EDE.0b013e318176bfcd

[6] Guo Y, Barnett AG, Yu W, Pan X, Ye X, et al (2011) A large change in temperature between neighbouring days increases the risk of mortality. PLoS One 6(2): e16511. 21311772 10.1371/journal.pone.0016511 PMC3032790

[7] Rocklöv J, Forsberg B (2010) The effect of high ambient temperature on the elderly population in three regions of Sweden. International journal of environmental research and public health 7(6): 2607–2619. 20644691 10.3390/ijerph7062607 PMC2905568

[8] Lin S, Luo M, Walker RJ, Liu X, Hwang SA, et al (2009) Extreme high temperatures and hospital admissions for respiratory and cardiovascular diseases. Epidemiology 20(5): 738. 19593155 10.1097/EDE.0b013e3181ad5522

[9] Ha J, Shin YS, Kim H (2011) Distributed lag effects in the relationship between temperature and mortality in three major cities in South Korea. Science of the Total Environment. 10.1016/j.scitotenv.2011.05.034 21683987

[10] Leung JY (2004) Climate Change in Hong Kong: Hong Kong Observatory.

[11] Vose RS, Easterling DR, Gleason B (2005) Maximum and minimum temperature trends for the globe: An update through 2004. Geophysical Research Letters.

[12] Li Q, Chen J (2008) Regional climate variations in south China related to global climate change and local urbanization. Proceedings of 16th IAHR-APD Congress and 3rd Symposium of IAHR-ISHS.

[13] Lim YH, Park AK, Kim H (2011) Modifiers of diurnal temperature range and mortality association in six Korean cities. Int J Biometeorol 56(1): 33–42. 21207069 10.1007/s00484-010-0395-0

[14] Liang WM, Liu WP, Kuo HW (2009) Diurnal temperature range and emergency room admissions for chronic obstructive pulmonary disease in Taiwan. Int J Biometeorol 53(1): 17–23. 18989710 10.1007/s00484-008-0187-y

[15] Song G, Chen G, Jiang L, Zhang Y, Zhao N, et al (2008) Diurnal temperature range as a novel risk factor for COPD death. Respirology 13(7): 1066–1069. 18922144 10.1111/j.1440-1843.2008.01401.x

[16] Tong S, Ren C, Becker N (2010) Excess deaths during the 2004 heatwave in Brisbane, Australia. International journal of biometeorology 54(4): 393–400. 20049484 10.1007/s00484-009-0290-8

[17] Zanobetti A, Schwartz J (2008) Temperature and mortality in nine US cities. Epidemiology 19(4): 563. 18467963 10.1097/EDE.0b013e31816d652d PMC3722554

[18] Michelozzi P, Accetta G, De Sario M, D’Ippoliti D, Marino C, et al (2009) High temperature and hospitalizations for cardiovascular and respiratory causes in 12 European cities. American journal of respiratory and critical care medicine 179(5): 383. 19060232 10.1164/rccm.200802-217OC

[19] Rocklöv J, Forsberg B (2008) The effect of temperature on mortality in Stockholm 1998–2003: A study of lag structures and heatwave effects. Scandinavian journal of public health 36(5): 516–523. 18567653 10.1177/1403494807088458

[20] Roberts S, Martin MA (2007) A distributed lag approach to fitting non-linear dose-response models in particulate matter air pollution time series investigations. Environmental research 104(2): 193–200. 17362914 10.1016/j.envres.2007.01.009

[21] Schwartz J (2000) Harvesting and long term exposure effects in the relation between air pollution and mortality. Am J Epidemiol 151(5): 440–448. 10707911 10.1093/oxfordjournals.aje.a010228

[22] Gasparrini A, Armstrong B, Kenward MG (2010) Distributed lag non-linear models. Stat Med 29(21): 2224–2234. 20812303 10.1002/sim.3940 PMC2998707

[23] Zeger SL, Dominici F, Samet J (1999) Harvesting-resistant estimates of air pollution effects on mortality. Epidemiology 10(2): 171–175. 10069254

[24] Schwartz J (2000) The distributed lag between air pollution and daily deaths. Epidemiology 11(3): 320. 10784251 10.1097/00001648-200005000-00016

[25] WHO (2010) International Statistical Classification of Diseases and Related Health Problems 10th Revision. http://apps.who.int/classifications/icd10/browse/2010/en. Accessed in March 7th, 2012.

[26] Hastie TJ, Tibshirani RJ (1990) Generalized additive models: Chapman & Hall/CRC. 10.1177/096228029500400302 8548102

[27] Armstrong B (2006) Models for the relationship between ambient temperature and daily mortality. Epidemiology 17(6): 624. 17028505 10.1097/01.ede.0000239732.50999.8f

[28] Anderson BG, Bell ML (2009) Weather-related mortality: how heat, cold, and heat waves affect mortality in the United States. Epidemiology 20(2): 205. 19194300 10.1097/EDE.0b013e318190ee08 PMC3366558

[29] D’Ippoliti Daniela MP, de’Donato Francesca MB, Klea K, Ursula K, Antonis A, et al (2010) The impact of heat waves on mortality in 9 European cities: results from the EuroHEAT project. Environmental Health 9(1): 37–45. 20637065 10.1186/1476-069X-9-37 PMC2914717

[30] Huynen MM, Martens P, Schram D, Weijenberg MP, Kunst AE (2001) The impact of heat waves and cold spells on mortality rates in the Dutch population. Environmental health perspectives 109(5): 463. 11401757 10.1289/ehp.01109463 PMC1240305

[31] Kan H, London SJ, Chen H, Song G, Chen G, et al (2007) Diurnal temperature range and daily mortality in Shanghai, China. Environ Res 103(3): 424–431. 17234178 10.1016/j.envres.2006.11.009

[32] Chen G, Zhang Y, Song G, Jiang L, Zhao N, et al (2007) Is diurnal temperature range a risk factor for acute stroke death? Int J Cardiol 116(3): 408–409. 16863670 10.1016/j.ijcard.2006.03.067

[33] Cao J, Cheng Y, Zhao N, Song W, Jiang C, et al (2009) Diurnal temperature range is a risk factor for coronary heart disease death. J Epidemiol 19(6): 328–332. 19749499 10.2188/jea.JE20080074 PMC3924102

[34] Tam WW, Wong TW, Chair SY, Wong AH (2009) Diurnal temperature range and daily cardiovascular mortalities among the elderly in Hong Kong. Arch Environ Occup Health 64(3): 202–206. 19864223 10.1080/19338240903241192

[35] Keatinge WR, Donaldson GC, Cordioli E, Martinelli M, Kunst AE, et al (2000) Heat related mortality in warm and cold regions of Europe: observational study. Bmj 321(7262): 670–673. 10987770 10.1136/bmj.321.7262.670 PMC27480

[36] Brazdil R, Budikova M, Auer I, Böhm R, Cegnar T, et al (1996) Trends of maximum and minimum daily temperatures in central and southeastern Europe. International journal of climatology 16(7): 765–782.

[37] Karl TR, Jones PD, Knight RW, Kukla G, Plummer N, et al.. (1993) Asymmetric trends of daily maximum and minimum temperature. Papers in Natural Resources: 185.

[38] Klein Tank A, Peterson T, Quadir D, Dorji S, Zou X, et al (2006) Changes in daily temperature and precipitation extremes in central and south Asia. J Geophys Res 111(D16105): 1–20.20411040

[39] Easterling DR, Horton B, Jones PD, Peterson TC, Karl TR, et al (1997) Maximum and minimum temperature trends for the globe. Science 277(5324): 364–367.

[40] Schneider A, Schuh A, Maetzel FK, Rückerl R, Breitner S, et al (2008) Weather-induced ischemia and arrhythmia in patients undergoing cardiac rehabilitation: another difference between men and women. International journal of biometeorology 52(6): 535–547. 18228048 10.1007/s00484-008-0144-9

[41] Carder M, McNamee R, Beverland I, Elton R, Cohen G, et al (2005) The lagged effect of cold temperature and wind chill on cardiorespiratory mortality in Scotland. Occupational and environmental medicine 62(10): 702–710. 16169916 10.1136/oem.2004.016394 PMC1740864

[42] Medina-Ramon M, Schwartz J (2007) Temperature, Temperature Extremes, and Mortality: A Study of Acclimatization and Effect Modification in 50 United States Cities. Occup Environ Med. 10.1136/oem.2007.033175 PMC2095353 17600037

[43] Braga AL, Zanobetti A, Schwartz J (2001) The time course of weather-related deaths. Epidemiology 12(6): 662–667. 11679794 10.1097/00001648-200111000-00014

[44] Basu R, Malig B (2011) High ambient temperature and mortality in California: exploring the roles of age, disease, and mortality displacement. Environ Res 111(8): 1286–1292. 21981982 10.1016/j.envres.2011.09.006

