# Supplementary figures and images for: Lagged Effect of Diurnal Temperature Range on Mortality in a Subtropical Megacity of China
Source: PLoS One. 2013 Feb 6;8(2):e55280. doi: 10.1371/journal.pone.0055280 (PMC3566202; doi:10.1371/journal.pone.0055280)

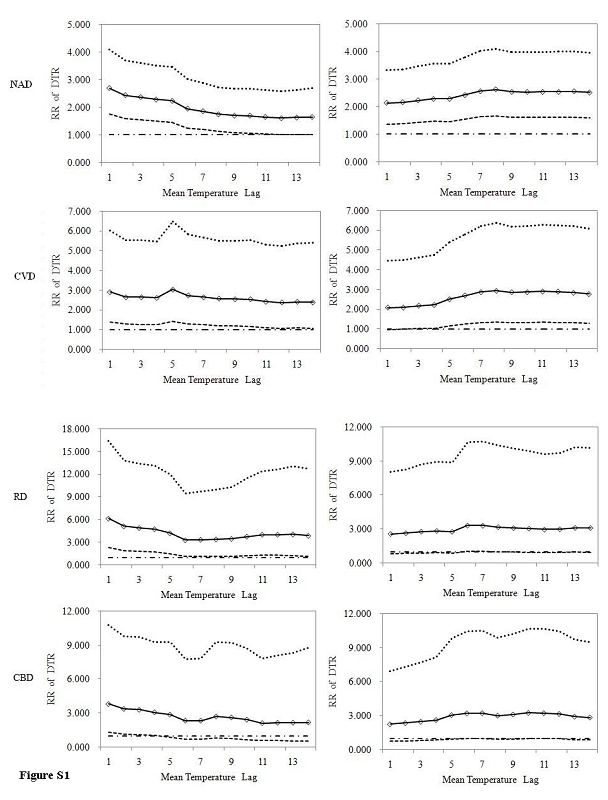

Supplement: Figure S1 — Association between DTR and mortality with adjustment of the maximum distributed lag days of mean temperature, 2006–2008. (TIF) [file pone.0055280.s001.tif]

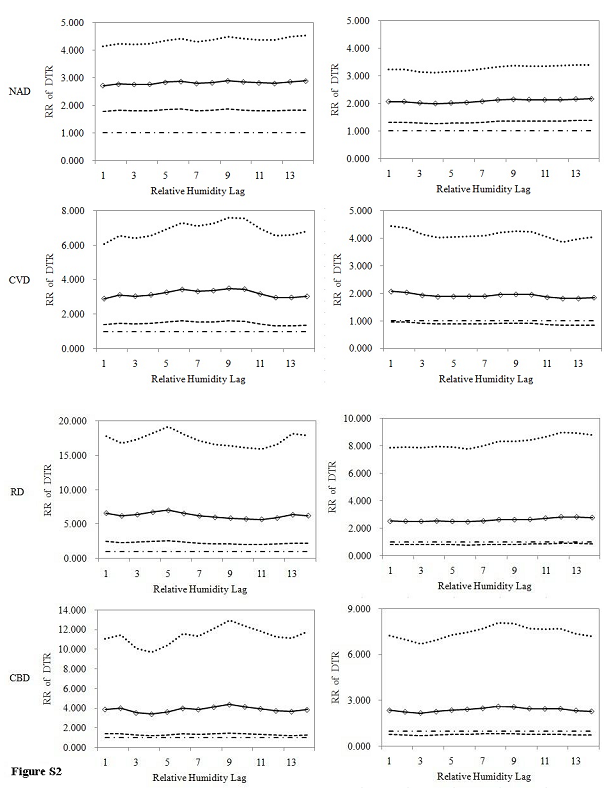

Supplement: Figure S2 — Association between DTR and mortality with adjustment of the maximum distributed lag days of relative humidity, 2006–2008. (TIF) [file pone.0055280.s002.tif]

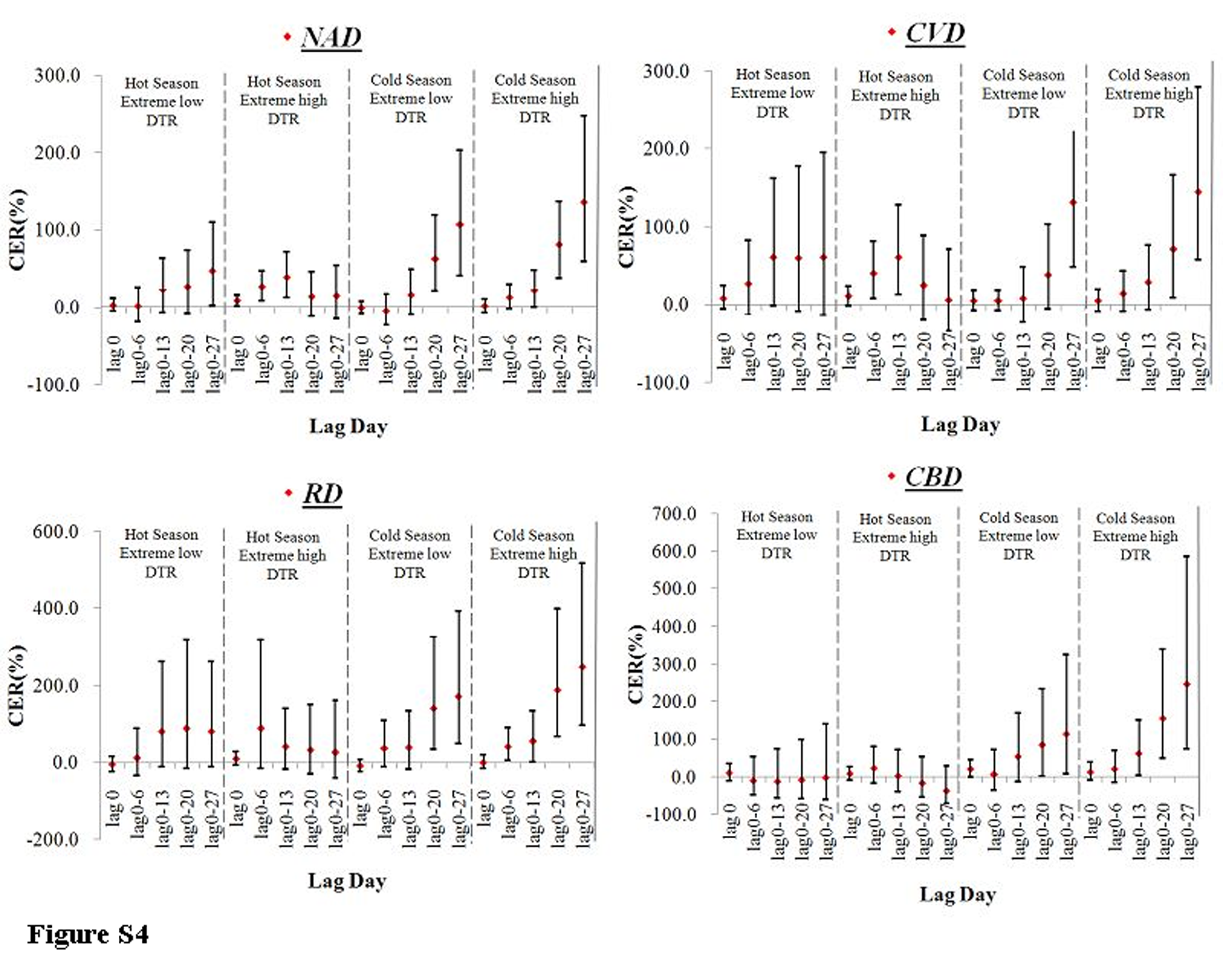

Supplement: Figure S4 — Effects of extreme low and high DTR on disease-specific deaths in hot and cold seasons at different lag times. The 8°C of DTR was selected as the reference, which was regarded as minimal mortality; Select selected lower than 2.3°C (2.5th percentile) and higher than 13.8°C (97.5th percentile) as the extreme low and high DTRs. (TIF) [file pone.0055280.s004.tif]

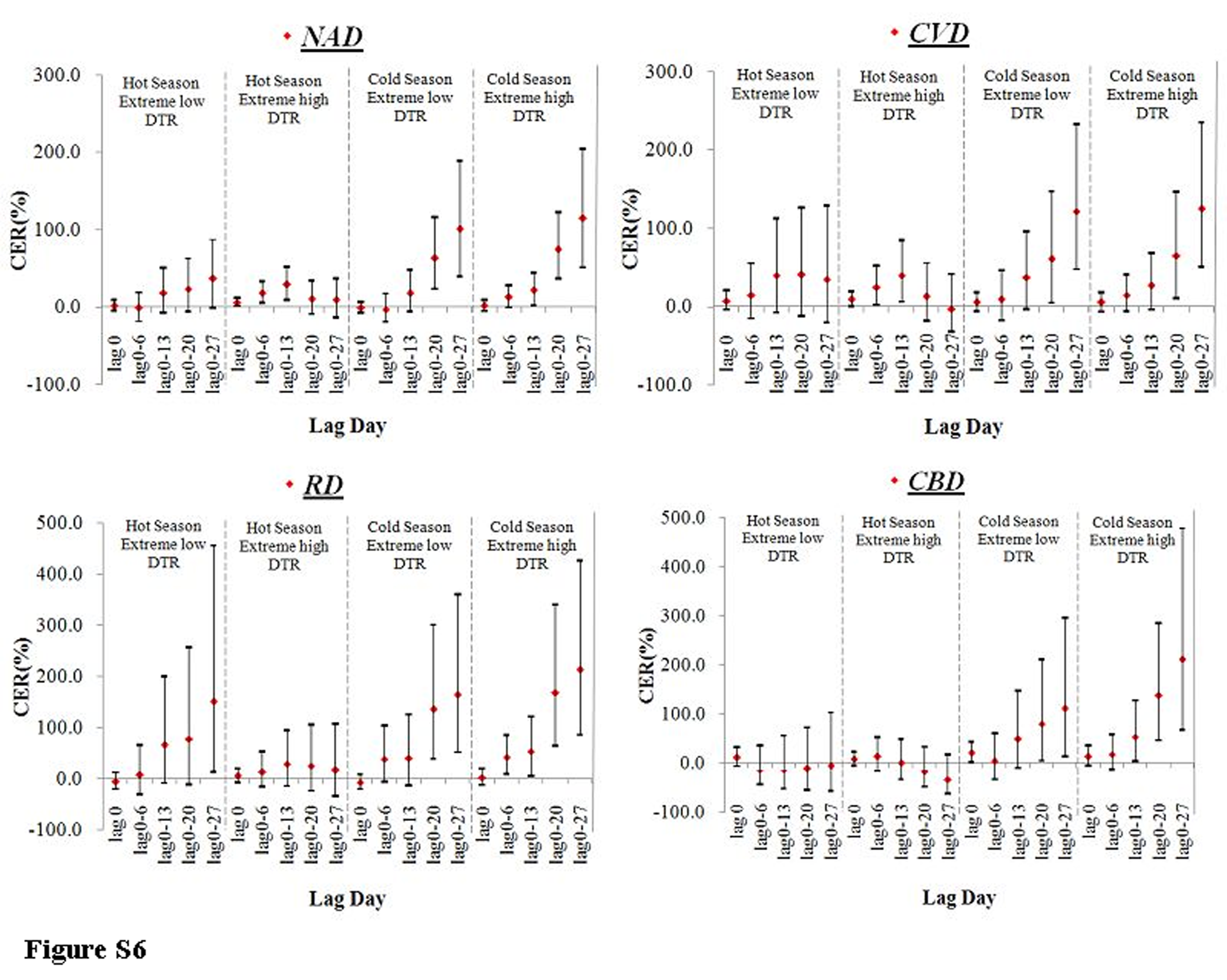

Supplement: Figure S6 — Effects of extreme low and high DTR on disease-specific deaths in hot and cold seasons at different lag times. The 8°C of DTR was selected as the reference, which was regarded as minimal mortality; Select selected lower than 3.1°C (5th percentile) and higher than 12.6°C (95th percentile) as the extreme low and high DTRs. (TIF) [file pone.0055280.s006.tif]
