# Supplementary material for: Lagged Effect of Diurnal Temperature Range on Mortality in a Subtropical Megacity of China
Source: PLoS One. 2013 Feb 6;8(2):e55280. doi: 10.1371/journal.pone.0055280 (PMC3566202; doi:10.1371/journal.pone.0055280)
Supplement: Table S1 — The CERs of different DTRs on mortality along 27 lag days for the full year. The type of SO2 and NO2 was considered as nonlinear in the DLNM. (DOC) [file pone.0055280.s008.doc]

# Table S1: The CERs of different DTRs on mortality along 27 lag days for the full year

| DTR  structures^*^ | CERs (95%CI) | | | |
| --- | --- | --- | --- | --- |
|  | NAD (%) | CVD (%) | RD (%) | CBD (%) |
| 1.7℃ | **140.9(55.6-273.2)** | **255.9(59.0-696.7)** | **369.3(65.5-12.3)** | **312.7(44.5-10.8)** |
| 5.5 ℃ | -7.8(-29.4-20.4) | 50.4(-3.2-133.6) | -15.5(-52.3-49.8) | -6.5(-48.5-69.8) |
| 7.6 ℃ | 3.1(-6.4-13.8) | 15.2(-3.3-37.2) | -0.8(-19.8-22.7) | 2.6(-19.0-29.9) |
| 9.2 ℃ | 2.0(-11.6-17.6) | 19.8(-1.6-45.7) | 18.7(-8.5-53.9) | 23.3(-6.4-62.5) |
| 14.5℃ | **128.4(45.8-257.7)** | 87.1(-20.8-342.4) | 249.4(9.9-10.1) | 118.4(-29.5-576.8) |

**^*^**1.7 ℃, 5.5 ℃, 7.6 ℃, 9.2 ℃ and 14.5 ℃ represent the 1^st^ percentile, 25^th^ percentile, 50^th^ percentile, 75^th^ percentile and 99^th^ percentile of DTR in Guangzhou, respectively. The 8 ℃ of DTR was selected as the reference. The type of SO2 and NO2 was considered as nonlinear in the DLNM.
